# Rod Angulation Relationship with Thoracic Kyphosis after Adolescent Idiopathic Scoliosis Posterior Instrumentation

**DOI:** 10.3390/children11010029

**Published:** 2023-12-26

**Authors:** Louis Boissiere, Anouar Bourghli, Fernando Guevara-Villazon, Ferran Pellisé, Ahmet Alanay, Frank Kleinstück, Javier Pizones, Cécile Roscop, Daniel Larrieu, Ibrahim Obeid

**Affiliations:** 1 ELSAN, Polyclinique Jean Villar, 53 Avenue Maryse Bastié, 33520 Bruges, France; 2 Spine Surgery Department, King Faisal Specialist Hospital and Research Center, Riyadh 11211, Saudi Arabia; 3 Spine Surgery Unit, Hospital Universitario Val Hebron, 08035 Barcelona, Spain; 4 Department of Orthopaedics and Traumatology, Acibadem University School of Medicine, Istanbul 34750, Turkey; 5 Research and Development, Schulthess Klinik, 8008 Zurich, Switzerland; 6 Spine Surgery Unit, Hospital Universitario La Paz, 28046 Madrid, Spain; 7 Spine Surgery Unit, CHU Pellegrin, 33076 Bordeaux, France

**Keywords:** adolescent idiopathic scoliosis, rod contour, thoracic kyphosis, predictive medicine, surgical planning

## Abstract

Introduction: Surgery to correct spinal deformities in scoliosis involves the use of contoured rods to reshape the spine and correct its curvatures. It is crucial to bend these rods appropriately to achieve the best possible correction. However, there is limited research on how the rod bending process relates to spinal shape in adolescent idiopathic scoliosis surgery. Methods: A retrospective study was conducted using a prospective multicenter scoliosis database. This study included adolescent idiopathic scoliosis patients from the database who underwent surgery with posterior instrumentation covering the T4 to T12 segments. Standing global spine X-rays were used in the analysis. The sagittal Cobb angles between T5 and T11 were measured on the spine. Additionally, the curvature of the rods between T5 and T11 was measured using the tangent method. To assess the relationship between these measurements, the difference between the dorsal kyphosis (TK) and the rod kyphosis (RK) was calculated (ΔK = TK − RK). This study aimed to analyze the correlation between ΔK and various patient characteristics. Both descriptive and statistical analyses were performed to achieve this goal. Results: This study encompassed a cohort of 99 patients, resulting in a total of 198 ΔK measurements for analysis. A linear regression analysis was conducted, revealing a statistically significant positive correlation between the kyphosis of the rods and that of the spine (r = 0.77, *p* = 0.0001). On average, the disparity between spinal and rod kyphosis averaged 5.5°. However, it is noteworthy that despite this modest mean difference, there was considerable variability among the patients. In particular, in 84% of cases, the concave rod exhibited less kyphosis than the spine, whereas the convex rod displayed greater kyphosis than the spine in 64% of cases. It was determined that the primary factor contributing to the flattening of the left rod was the magnitude of the coronal Cobb angle, both before and after the surgical procedure. These findings emphasize the importance of considering individual patient characteristics when performing rod bending procedures, aiming to achieve the most favorable outcomes in corrective surgery. Conclusions: Although there is a notable and consistent correlation between the curvature of the spine and the curvature of the rods, it is important to acknowledge the substantial heterogeneity observed in this study. This heterogeneity suggests that individual patient factors play a significant role in shaping the outcome of spinal corrective surgery. Furthermore, this study highlights that more severe spinal curvatures in the frontal plane have an adverse impact on the shape of the rods in the sagittal plane. In other words, when the scoliosis curve is more pronounced in the frontal plane, it tends to influence the way the rods are shaped in the sagittal plane. This underscores the complexity of spinal deformities and the need for a tailored approach in surgical interventions to account for these variations among patients.

## 1. Introduction

The surgical management of adolescent idiopathic scoliosis (AIS) has evolved over the years, and the posterior approach instrumentation, correction, and fusion have emerged as the gold standard for treating this condition [1]. The success of this surgical procedure hinges on several key factors that play a crucial role throughout the operation. These factors include carefully selecting the appropriate fusion levels, achieving optimal rod bending in the sagittal plane, and executing precise reduction maneuvers [2].

The correction of the frontal plane deformity, which can be assessed by the measurement of the Cobb angle, has been a well-established part of scoliosis surgery for many years [3]. However, achieving sagittal plane correction is more challenging, especially when instrumentation of the thoracic spine is necessary [4]. One of the concerns during surgery is the potential for increasing thoracic kyphosis, which can impact the patient’s overall spinal alignment [5].

Scoliosis typically leads to the flattening of the spine in most cases [6,7], emphasizing the importance of achieving a harmonious balance in the instrumented portion of the spine. To ensure the best possible outcome, meticulous preoperative surgical planning is indispensable [8]. This planning process helps formulate the surgical strategy and establish radiological goals, providing guidance for the surgical team [9].

The success of scoliosis surgery depends on various factors, and achieving optimal rod bending is among the critical elements [10]. Previous research has explored the intricate connection between proper rod bending and the successful execution of surgical planning [11]. Additionally, numerous studies have delved into the correlation between rod curvature and spinal curvature, employing diverse measurement methods. These investigations have encompassed degenerative spine surgery [12], spine trauma [13], adult spinal deformity (ASD) surgery [14], and AIS surgery [15]. However, it is noteworthy that the relationship between rod and spine curvatures may exhibit variations among these studies.

In recent times, there has been a growing interest in the development of specialized custom-made rods to achieve ideal corrections [11]. These rods are custom manufactured based on preoperative planning and are expected to offer superior correction compared to traditional rods manually bent by the surgeon during the procedure [16]. However, it is essential to underscore that the benefits of these specialized rods remain unproven, and there is currently no definitive data supporting the notion that these ideal rods consistently produce the best correction. It is crucial to recognize that various factors come into play during surgery, including compression and distraction maneuvers and the inherent flexibility of the spine.

During reduction maneuvers, the rod undergoes mechanical stress, leading to gradual deformation. This deformation results in a different shape for the rod before and after reduction, with some studies reporting an average angular loss of 20 degrees during AIS surgery, particularly in the concave portion of the curve [17]. To mitigate the risk of rod deformation, the choice of rod material (titanium alloy or cobalt chrome) or rod diameter (5.5 to 6 mm) can also influence deformation. As of now, there is no conclusive evidence favoring one type of rod over another [18]. Most studies report equivalent outcomes in terms of correction percentage, consolidation, or breakage, irrespective of the rod type used.

Given the complexity of the factors involved, there is still a vast field of investigation to fully comprehend the precise impact of reduction maneuvers. This raises the critical question of the predictability of surgical outcomes. A multitude of studies will be required to address these complex issues comprehensively [19].

However, as an initial step, the purpose of this study is to assess the relationship between rod shape and spinal shape on the first postoperative radiography. The primary inquiry revolves around whether the final shape of the rod can independently predict the ultimate shape of the spine in the thoracic fused spine after AIS surgery. Additionally, we aim to identify any predictive factors that may influence this relationship.

## 2. Material and Methods

### 2.1. Design

This study adopted a retrospective design, making use of a prospective multicenter database that centered its focus on operated adolescents for scoliosis and Scheuermann disease. The database’s inclusion criteria encompassed individuals who had undergone surgery for AIS and Scheuermann kyphosis, all of whom were below 18 years old at the time of the initial assessment. Notably, the database did not include cases of congenital scoliosis.

From this extensive database, the study population consisted of patients who had undergone posterior fusion surgery for AIS. These patients were required to have a minimum follow-up period of 3 months, with their first postoperative X-ray serving as the baseline assessment. To maintain consistency and homogeneity in the study group, cases involving left thoracic major curves and Scheuermann kyphosis were excluded. The analysis focused on cases involving the fixation of spinal segments from T5 to T11.

In all instances, the upper instrumented vertebra (UIV) was positioned at T4 or higher, while the lower instrumented vertebra (LIV) was situated at T12 or lower. At the time of data extraction, the database included records for a total of 171 patients, of whom 72 patients were excluded from the subsequent analysis. Ultimately, this study included and analyzed data from 99 patients who met the specified criteria.

### 2.2. Surgery Technique

The surgical procedures were performed by multiple surgeons from four different spine centers, all following established standards for correcting adolescent idiopathic scoliosis. Notably, there were variations in the reduction techniques used across these centers. In all cases, posterior spinal pedicle screw instrumentation was utilized. To ensure surgical precision, neurophysiological monitoring was consistently applied throughout the procedures. The placement of pedicle screws was carried out using a combination of the freehand technique, fluoroscopy, or navigation, depending on the specific surgical site and timing.

In situations where the spine exhibited rigidity, posterior column osteotomies were conducted to enhance flexibility. Surgeons took into account the resulting thoracic kyphosis and lumbar lordosis when shaping the rods. To achieve the desired reduction, derotation and/or translation maneuvers were applied to one or both rods. In certain cases, additional in situ adjustments, such as over or under-contouring, along with interpedicular “compression-distraction” techniques, were employed to optimize the final construct.

This approach allowed for a comprehensive understanding of the surgical procedures performed across different centers while highlighting the variability in reduction techniques and the emphasis on precision throughout. It also underscored the importance of considering spine flexibility and adopting various maneuvers to achieve the desired correction during AIS surgery [6,20].

### 2.3. Data Collection and Radiographic Measurement

We collected demographic and radiographical data for our study. Radiographic analysis was performed using KEOPS^®^ software (www.keops-spine.fr) based in Paris, France. The measurement of thoracic kyphosis (TK) in the T5-T11 region was conducted using the Cobb method, which determines the angle between the line parallel to the upper endplate of T5 and the line parallel to the lower endplate of T11 [21].

**Figure 1:**
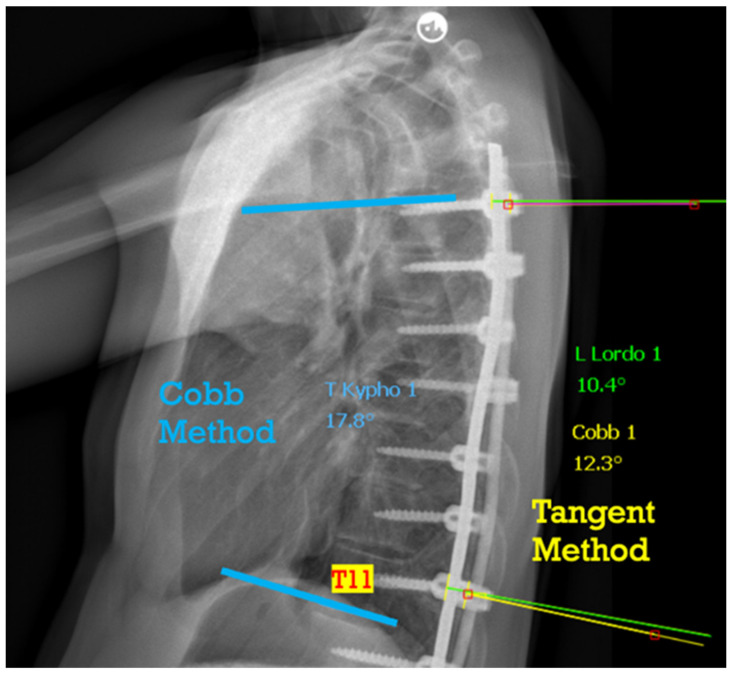
T5–T11 thoracic measurement with the Cob method and T5–T11 rod measurement with the tangent method.

Additionally, we measured left and right rod kyphosis (RK) in the T5–T11 segment using the tangent method. This measurement involved calculating the angle formed by the perpendicular line to the tangent of the rod at the T5 and T11 screw positions [14] (Figure 1). To assess the difference between TK and RK, we computed the value ΔK, which is obtained by subtracting RK from TK (ΔK = TK − RK). The gathered parameters are typically those regularly collected for assessing an AIS cohort, including age, gender, instrumented levels, rod specifications, and the primary Cobb angle measurement.

### 2.4. Statistical Analysis

We summarized the descriptive parameters of the population using means and standard deviations. To assess the relationship between T5–T11 thoracic kyphosis (TK) and rod kyphosis (RK), we employed Pearson’s correlation coefficient. Additionally, we graphed and measured the ΔK values. To investigate the associations between ΔK and other variables (preoperative and postoperative major Cobb angle, right and left rod), we conducted univariate analyses and calculated Pearson’s correlation coefficients. Statistical significance was determined if the “*p*” value was less than 0.05. All statistical analyses were conducted using IBM SPSS Statistics 23.0 (SPSS Inc., Chicago, IL, USA).

## 3. Results

We analyzed a total of 198 rods from 99 patients. Table 1 summarizes the demographic data (gender, age), surgical details (instrumented levels, implants, osteotomies), and radiographic measurements (Cobb angle, kyphosis, lordosis, rod curvature, and ΔK).

The mean SRS-22 score significantly improved from 3.69 (SD 0.67) in preoperative to 4.29 (SD 0.73) in postoperative.

The average correction of the major curve in the coronal plane amounted to 42.4%, reducing from an initial measurement of 62.6° (SD 12.8) to 26.6° (SD 9.4). In contrast, the T5-T11 thoracic kyphosis exhibited a flattening trend, decreasing from 25.1° (SD 16.4) to 23.7° (SD 8.6). However, upon conducting a paired sample T-test, this change was found to be statistically non-significant (*p* = 0.25). It is important to note that this study did not distinguish between hypokyphotic and hyperkyphotic thoracic curves.

**Figure 2:**
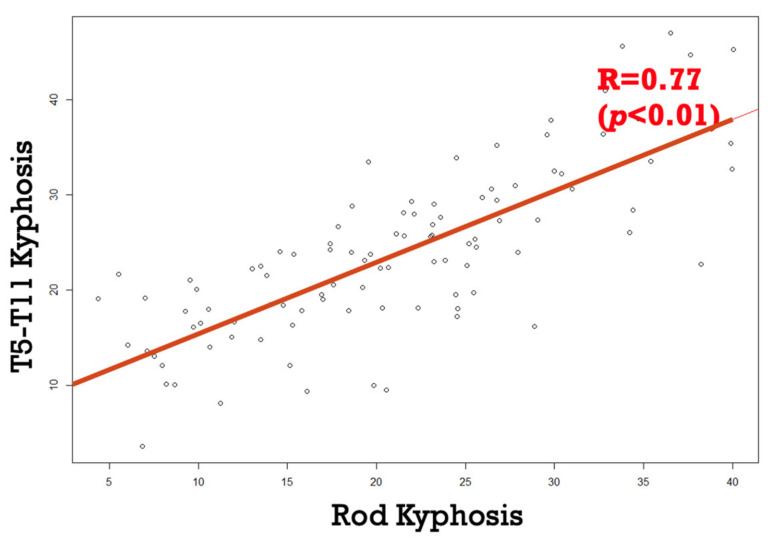
Strong correlation between rod and spine thoracic kyphosis.

The average measurement for thoracic kyphosis (TK) between T5 and T11 was 23.7° (SD 8.6), while the corresponding measurement for rod kyphosis (RK) was 20.9° (SD 8.9). A paired sample correlation analysis between T5-T11 RK and TK revealed consistent correlation values (R = 0.77, *p* < 0.01) (Figure 2).

**Figure 3:**
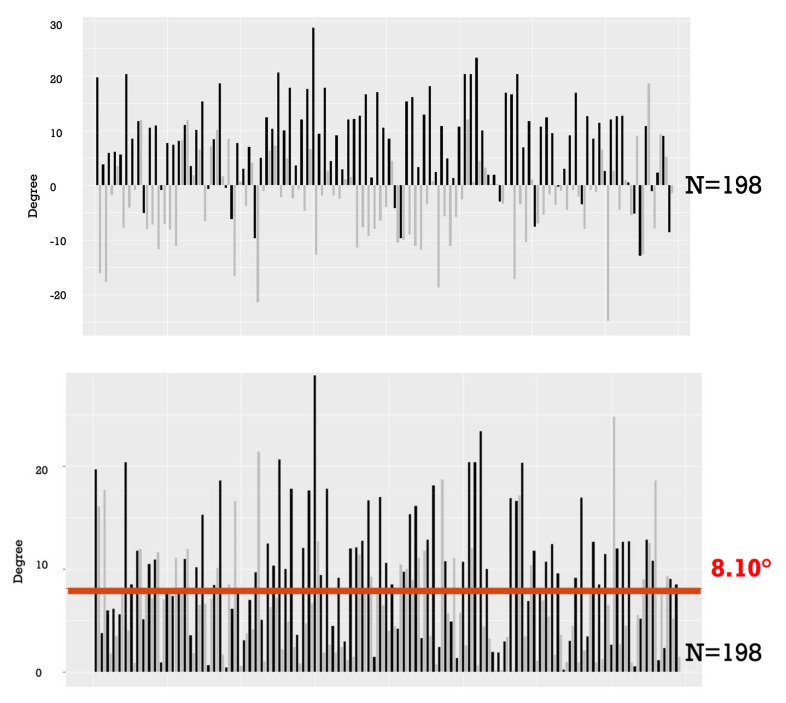
Numeric and absolute value of the difference between rod and spine kyphosis. The left rods are represented in black, while the right rods are shown in gray. The red line represents the mean average between the rod and spine kyphosis.

The difference between TK and RK amounted to an average of 8.10° (SD 5.84). Notably, 75% of the values exhibited higher TK than RK, indicating a positive ΔK. Furthermore, 63% of the patients displayed a difference, in absolute value, of ΔK greater than 5° (Figure 3).

**Figure 4:**
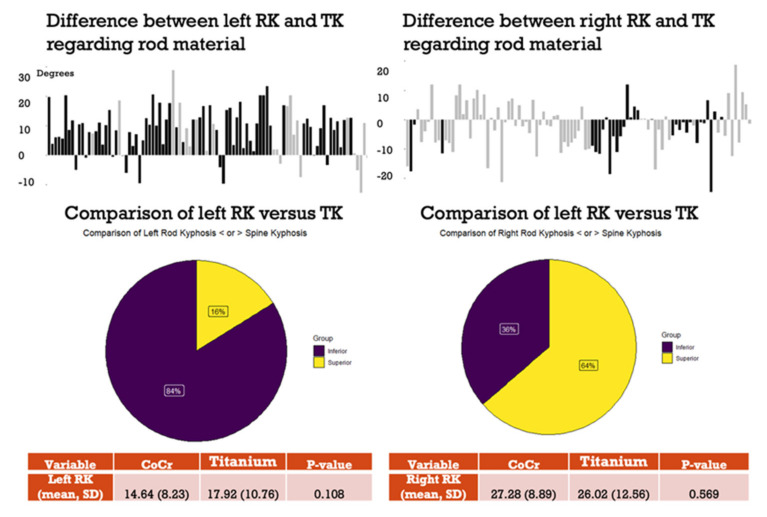
Differences between left and right rod and TK.

We conducted a comparison between the left rod, typically the concave rod, and the right convex rod. Our findings revealed that 84% of the left rods exhibited less kyphosis than thoracic kyphosis of the spine. Conversely, in the case of the right rods, 64% displayed greater kyphosis than the spine’s thoracic kyphosis. Importantly, no significant differences in kyphosis were observed between the two rod materials, cobalt chrome and titanium alloy (Figure 4).

**Figure 5:**
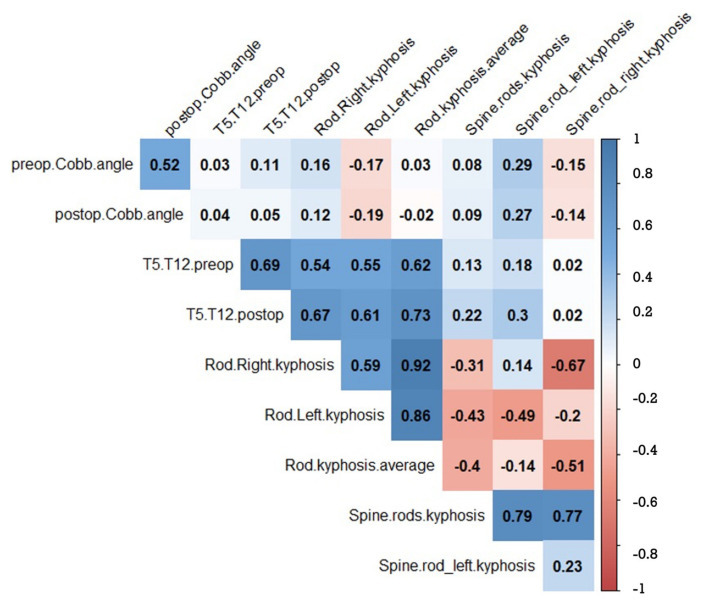
Significant correlation factors between preoperative, postoperative coronal Cobb angle, T5–T11 TK, and left and right RK. The blank squares are non-significant correlation factors.

Both preoperative and postoperative thoracic kyphosis (TK) exhibited a strong correlation with the kyphosis of the left and right rods. In particular, a larger preoperative or postoperative Cobb angle was associated with a flatter left rod (correlation coefficients: r = 0.29 and r = 0.27, respectively). This correlation remained consistently strong when comparing the difference between preoperative and postoperative T5–T11 TK with the mean rod kyphosis (left and right) or the individual left and right rod kyphosis (Figure 5).

## 4. Discussion

Despite the dearth of studies exploring the factors influencing surgical predictability, this study affirms a robust linear correlation between rod curvature and spine curvature. This underscores the critical role of proper rod bending in achieving desired postoperative results [22]. The sagittal plane restoration in spinal deformity surgery presents an intriguing challenge due to the inherent curvatures of the spine. The introduction of the Lenke classification brought sagittal plane analysis in AIS to the forefront and encouraged the adoption of various surgical techniques aimed at improving thoracic kyphosis [23,24,25]. The exact relationship between the rod and spine curvatures remains unclear. This study supports the fact that bending the rod will impact the final shape of the spine. 

However, it is crucial to note that more than half of the rods deviated by over 5° from the expected thoracic kyphosis. This deviation raises valid questions about the rod’s ability to reliably predict the final correction. The difference observed between concave and convex rods serves as a stark reminder that scoliosis is a three-dimensional deformity. Both rods play distinct roles in the reduction process, and their bending is influenced by both the desired final spinal shape and the mechanical stresses encountered during reduction maneuvers [26,27].

While AIS surgery employs very stiff rods, concave left rods have a propensity to flatten significantly. To counteract this effect, surgeons often recommend overbending the concave rod before commencing the reduction process [17]. Interestingly, our study did not reveal significant differences between titanium and cobalt chrome rods, implying that rod material may not exert a substantial influence on surgical outcomes. This observation aligns with existing literature that generally does not find material-dependent differences [28].

The parameter most closely correlated with rod flattening is the major Cobb angle in the coronal plane. A larger Cobb angle typically signifies a stiffer spine and a more substantial correction. However, much like previous studies, we encountered challenges in directly comparing the loss of kyphosis in rods before and after placement. Nevertheless, we concur that increasing the curvature of the concave rod remains essential. Nevertheless, the substantial variability observed between rod curvature and thoracic kyphosis suggests that relying solely on rod centering may be insufficient. Instead, the shape and rigidity of the spine are likely pivotal in determining the final rod shape.

In our study, the T5–T11 segment exhibited less kyphosis postoperatively compared to preoperative measurements. It is worth noting that our analysis intentionally focused on a specific spinal segment. We aimed to evaluate the relationship between rod and spine curvature more directly, rather than quantifying changes in kyphosis between pre- and postoperative states, a common approach in evaluating T4–T12 or T2–T12 kyphosis. The reason is to concentrate our attention on the completely fused part of the spine so that the results cannot be influenced by the adjacent mobile junctional segments.

As with any study, ours has several limitations. It is inherently retrospective and involves a variety of surgical strategies and techniques. Importantly, our study does not seek to evaluate the surgical outcomes of AIS correction; instead, its primary focus is on understanding the intricate relationship between the rod and spine curvature. Additionally, we must acknowledge the inherent complexity of scoliosis, a three-dimensional deformity being analyzed through two-dimensional parameters. This complexity necessitates further investigations aimed at refining predictive factors for final radiological outcomes. Beyond angular correction, elements such as transitional zones [29], apex location, and curve magnitude all warrant in-depth exploration to advance our understanding of AIS surgical correction.

## 5. Conclusions

For AIS correction surgery, the objective is often to increase thoracic kyphosis. The rod must be contoured appropriately and is strongly correlated with the spine’s shape. Despite this correlation, in many cases, we observe significant variability between the curvature of the spine and the curvature of the rod. Multiple factors can explain this variability, but the anatomical factors linked to scoliosis itself (major Cobb angle) seem to be more impactful than surgical factors such as rod material. With these observations, it currently appears challenging to believe that a so-called ‘ideal’ rod would lead to a better correction.

## Figures and Tables

**Table 1:** Demographic and radiologic descriptive data.

Variable	Mean (Standard Deviation)	Cases
Gender		86 F, 13 M
Age	14 (1.6)	
Posterior Instrumented Levels	11 (2.6)	
Number of Implants	17 (5.1)	
Posterior Column Osteotomy		15 (14.1%)
Preoperative Major Cobb Angle	62.6° (12.8)	
Postoperative Major Cobb Angle	26.6° (9.4)	
Preoperative T5–T11 Kyphosis	25.1° (16.4)	
Postoperative T5–T11 Kyphosis	23.7° (8.6)	
Mean Rod Curvature (T5–T11)	20.9° (8.9)	
Preoperative Lumbar Lordosis	59.4° (23.1)	
Postoperative Lumbar Lordosis	58.3° (10.4)	
T5–T11 ΔK	2.7° (6)	
Mean Absolute T5–T11 ΔK	5.5° (3.6)	

## Data Availability

The data presented in this study are available on request from the corresponding author. The data are not publicly available due to all data are part of the ESSG registry.
